# Supplementary material for: Exposure to the 1959–1961 Chinese famine and risk of non-communicable diseases in later life: A life course perspective
Source: PLOS Glob Public Health. 2023 Aug 16;3(8):e0002161. doi: 10.1371/journal.pgph.0002161 (PMC10431657; doi:10.1371/journal.pgph.0002161)
Supplement: S1 Text — (DOCX) [file pgph.0002161.s001.docx]

**S1 Text. Categorization of Life Stages based on Erikson’s Developmental Stages.**

We assigned (1) participants born in 1959 to “the fetal stage (newborn)”, (2) participants born in 1958 to “the infancy stage (1 year)”, (3) participants born between 1956 and 1957 to “the early childhood stage (2-3 years)”, (4) participants born between 1954 and 1955 to “the preschool stage (4-5 years)”, (5) participants born between 1948 and 1953 to “the school age stage (6-11 years)”, (6) participants born between 1941 and 1947 to “the adolescence stage (12-18 years)”, and (7) participants born between 1919 and 1940 to “the young adulthood stage (19-40 years)”.
