# Supplementary material for: Exposure to the 1959–1961 Chinese famine and risk of non-communicable diseases in later life: A life course perspective
Source: PLOS Glob Public Health. 2023 Aug 16;3(8):e0002161. doi: 10.1371/journal.pgph.0002161 (PMC10431657; doi:10.1371/journal.pgph.0002161)
Supplement: S1 Table — (DOCX) [file pgph.0002161.s002.docx]

**S1 Table.** **Associations between exposure to the 1959-1961 Chinese famine, life stage, and later-life NCDs (based on life stages commonly used in major global policies and strategies).**

|  | 1959.01.01 as reference date | |  | 1960.01.01 as reference date | |  | 1961.01.01 as reference date | |
| --- | --- | --- | --- | --- | --- | --- | --- | --- |
|  | IRRs | 95% CI |  | IRRs | 95% CI |  | IRRs | 95% CI |
| Unexposed (control group) | 1.00 | Reference |  | 1.00 | Reference |  | 1.00 | Reference |
| Exposed in-utero | 1.90^***^ | 1.70–2.12 |  | 2.00^***^ | 1.76–2.27 |  | 2.03^***^ | 1.83–2.25 |
| Exposed in “first 1,000 days” (0-2 years)^1^ | 1.86^***^  (1.95^***^) | 1.73–2.00  (1.67–2.29) |  | 1.77^***^  (1.80^***^) | 1.63–1.91  (1.56–2.07) |  | 1.83^***^  (2.13^***^) | 1.68–1.99  (1.79–2.54) |
| Exposed in pre-school age (3-5 years) | 1.56^***^ | 1.47–1.66 |  | 1.61^***^ | 1.51–1.72 |  | 1.64^***^ | 1.54–1.76 |
| Exposed in primary school age (6-9 years) | 1.45^***^ | 1.37–1.54 |  | 1.44^***^ | 1.36–1.52 |  | 1.41^***^ | 1.34–1.49 |
| Exposed in adolescence (10-18 years) | 1.08^**^ | 1.03–1.14 |  | 1.12^***^ | 1.07–1.18 |  | 1.14^***^ | 1.09–1.20 |
| Exposed in young adulthood (19-23 years) | 0.66^***^ | 0.61–0.71 |  | 0.72^***^ | 0.67–0.77 |  | 0.75^***^ | 0.70–0.80 |
| Exposed in adulthood (24-40 years) | 0.40^***^ | 0.37–0.44 |  | 0.42^***^ | 0.38–0.45 |  | 0.43^***^ | 0.40–0.46 |
| Number of participants | 11,094 |  |  | 11,361 | |  | 11,823 |  |
| Number of observations | 39,337 |  |  | 40,297 | |  | 41,926 |  |
| *Note.* IRRs = Incidence Rate Ratios. ^*^*p<* .05, ^**^*p<* .01, ^***^*p<* .001 | | | | | |  |  |  |

Adjusted for age, sex, later-life residence, marital status, current working status, childhood family financial status, education, household income, number of diseases in childhood, and number of diseases in adulthood.

^1^ Numbers in parentheses refer to the IRRs and 95% CI for participants exposed in the 0-6 months.
