# Supplementary material for: Exposure to the 1959–1961 Chinese famine and risk of non-communicable diseases in later life: A life course perspective
Source: PLOS Glob Public Health. 2023 Aug 16;3(8):e0002161. doi: 10.1371/journal.pgph.0002161 (PMC10431657; doi:10.1371/journal.pgph.0002161)
Supplement: S2 Table — (DOCX) [file pgph.0002161.s003.docx]

**S2 Table.** **Associations between exposure to the 1959-1961 Chinese famine, life stage, and later-life NCDs (based on Erikson’s developmental stages).**

|  | 1959.01.01 as reference date | |  | 1960.01.01 as reference date | | |  | 1961.01.01 as reference date | | |
| --- | --- | --- | --- | --- | --- | --- | --- | --- | --- | --- |
|  | IRRs | 95% CI |  | IRRs | 95% CI | |  | IRRs | | 95% CI |
| Unexposed (control group) | 1.00 | Reference |  | 1.00 | Reference | |  | 1.00 | | Reference |
| Exposed at the fetal stage (newborn) | 1.86^***^ | 1.64–2.12 |  | 1.77^***^ | 1.56–2.01 | |  | 2.02^***^ | | 1.76–2.31 |
| Exposed at the infancy stage (1 year) | 1.73^***^ | 1.56–1.92 |  | 1.78^***^ | 1.58–2.00 | |  | 1.73^***^ | | 1.55–1.94 |
| Exposed at the early childhood stage (2-3 years) | 1.72^***^ | 1.60–1.86 |  | 1.74^***^ | 1.61–1.88 | |  | 1.65^***^ | | 1.52–1.79 |
| Exposed at the preschool stage (4-5 years) | 1.51^***^ | 1.41–1.61 |  | 1.49^***^ | 1.39–1.60 | |  | 1.60^***^ | | 1.49–1.73 |
| Exposed at the school age stage (6-11 years) | 1.37^***^ | 1.30–1.45 |  | 1.38^***^ | 1.31–1.45 | |  | 1.37^***^ | | 1.30–1.44 |
| Exposed at the adolescence stage (12-18 years) | 1.03 | 0.97–1.09 |  | 1.06^*^ | 1.01–1.12 | |  | 1.08^**^ | | 1.03–1.14 |
| Exposed at the young adulthood stage (19-40 years) | 0.55^***^ | 0.51–0.58 |  | 0.58^***^ | 0.54–0.62 | |  | 0.60^***^ | | 0.57–0.64 |
| Number of participants | 10,855 | |  | 11,193 | | |  | 11,532 | | |
| Number of observations | 38,495 | |  | 39,701 | | |  | 40,891 | | |
| *Note.* IRRs = Incidence Rate Ratios. ^*^*p<* .05, ^**^*p<* .01, ^***^*p<* .001 | | |  |  | |  |  | |  |  |

Adjusted for age, sex, later-life residence, marital status, current working status, childhood family financial status, education, household income, number of diseases in childhood, and number of diseases in adulthood.
