# Supplementary material for: Exposure to the 1959–1961 Chinese famine and risk of non-communicable diseases in later life: A life course perspective
Source: PLOS Glob Public Health. 2023 Aug 16;3(8):e0002161. doi: 10.1371/journal.pgph.0002161 (PMC10431657; doi:10.1371/journal.pgph.0002161)
Supplement: S3 Table — (DOCX) [file pgph.0002161.s004.docx]

**S3 Table.** **Associations between severity of exposure to the 1959-1961 Chinese famine and later-life NCDs.**

|  | IRRs | 95% CI |
| --- | --- | --- |
| Moderate famine exposure | 1.18^***^ | 1.09–1.28 |
| Severe famine exposure | 1.24^***^ | 1.17–1.32 |
| Age | 1.99^***^ | 1.91–2.08 |
| Sex (-0.5 = *male*, +0.5 = *female*) | 1.16^***^ | 1.10–1.22 |
| Later-life residence | 0.90^***^ | 0.85–0.95 |
| Marital status | 1.02 | 0.97–1.08 |
| Current working status | 0.91^***^ | 0.88–0.94 |
| Childhood family financial status | 1.01 | 0.99–1.04 |
| Upper secondary or vocational education | 1.05 | 0.94–1.17 |
| Tertiary education | 1.13 | 0.91–1.40 |
| Income decile (1 = *bottom 10%*, 10 = *top 10%*) | 1.00 | 0.99–1.01 |
| Number of diseases in childhood | 1.12^**^ | 1.04–1.20 |
| Number of diseases in adulthood | 1.24^***^ | 1.19–1.28 |
| Number of participants | 4,082 |  |
| Number of observations | 14,471 |  |

*Note.* IRRs = Incidence Rate Ratios. ^*^*p<* .05, ^**^*p<* .01, ^***^*p<* .001
