# Supplementary material for: Exposure to the 1959–1961 Chinese famine and risk of non-communicable diseases in later life: A life course perspective
Source: PLOS Glob Public Health. 2023 Aug 16;3(8):e0002161. doi: 10.1371/journal.pgph.0002161 (PMC10431657; doi:10.1371/journal.pgph.0002161)
Supplement: S4 Table — (DOCX) [file pgph.0002161.s005.docx]

**S4 Table.** **Sex-specific associations between exposure to the 1959-1961 Chinese famine and later-life NCDs.**

|  | IRRs | 95% CI |
| --- | --- | --- |
| Famine exposure | 1.14^***^ | 1.09–1.19 |
| Sex (-0.5 = *male*, +0.5 = *female*) | 1.17^***^ | 1.08–1.27 |
| Famine exposure * Sex | 0.98 | 0.90–1.07 |
| Age | 1.99^***^ | 1.94–2.04 |
| Later-life residence | 0.93^***^ | 0.90–0.97 |
| Current marital status | 1.03^*^ | 1.01–1.07 |
| Current working status | 0.91^***^ | 0.89–0.93 |
| Childhood family financial status | 1.04^***^ | 1.02–1.05 |
| Upper secondary or vocational education | 1.00 | 0.94–1.06 |
| Tertiary education | 1.12 | 0.97–1.29 |
| Income decile (1 = *bottom 10%*, 10 = *top 10%*) | 1.00 | 0.99–1.00 |
| Number of diseases in childhood | 1.13^***^ | 1.08–1.19 |
| Number of diseases in adulthood | 1.23^***^ | 1.20–1.25 |
| Number of participants | 11,094 |  |
| Number of observations | 39,337 |  |

*Note.* IRRs = Incidence Rate Ratios. ^*^*p<* .05, ^**^*p<* .01, ^***^*p<* .001
