# Supplementary material for: Exposure to the 1959–1961 Chinese famine and risk of non-communicable diseases in later life: A life course perspective
Source: PLOS Glob Public Health. 2023 Aug 16;3(8):e0002161. doi: 10.1371/journal.pgph.0002161 (PMC10431657; doi:10.1371/journal.pgph.0002161)
Supplement: S5 Table — (DOCX) [file pgph.0002161.s006.docx]

**S5 Table. Age of NCDs onset among participants exposed to famine at different life stage.**

|  | Mean age of NCDs onset | |
| --- | --- | --- |
| In-utero | 40.0 (*SD* = 12.5) |  |
| The “first 1,000 days” (0-2 years) | 42.4 (*SD* = 13.0) |  |
| Pre-school (3-5 years) | 44.0 (*SD* = 13.4) |  |
| Primary school (6-9 years) | 45.7 (*SD* = 15.0) |  |
| Adolescence (10-18 years) | 49.7 (*SD* = 16.5) |  |
| Young adulthood (19-23 years) | 56.0 (*SD* = 18.1) |  |
| Adulthood (24-40 years) | 62.2 (*SD* = 18.6) |  |
