# Supplementary material for: Exposure to the 1959–1961 Chinese famine and risk of non-communicable diseases in later life: A life course perspective
Source: PLOS Glob Public Health. 2023 Aug 16;3(8):e0002161. doi: 10.1371/journal.pgph.0002161 (PMC10431657; doi:10.1371/journal.pgph.0002161)
Supplement: S6 Table — (DOCX) [file pgph.0002161.s007.docx]

**S6 Table. Associations between severity of the 1959-1961 Chinese famine and later-life NCDs.**

|  | IRRs | 95% CI |
| --- | --- | --- |
| Exposed in moderately affected areas | 1.20^***^ | 1.14–1.26 |
| Exposed in severely affected areas | 1.11^***^ | 1.06–1.17 |
| Age | 1.99^***^ | 1.94–2.05 |
| Sex (-0.5 = *male*, +0.5 = *female*) | 1.16^***^ | 1.12–1.20 |
| Later-life residence | 0.93^***^ | 0.90–0.97 |
| Marital status | 1.03 | 1.00–1.07 |
| Current working status | 0.91^***^ | 0.89–0.93 |
| Childhood family financial status | 1.03^***^ | 1.02–1.05 |
| Upper secondary or vocational education | 1.00 | 0.94–1.07 |
| Tertiary education | 1.13 | 0.98–1.30 |
| Income decile (1 = *bottom 10%*, 10 = *top 10%*) | 1.00 | 0.99–1.00 |
| Number of diseases in childhood | 1.14^***^ | 1.09–1.20 |
| Number of diseases in adulthood | 1.22^***^ | 1.20–1.25 |
| Number of participants | 10,840 |  |
| Number of observations | 38,420 |  |

*Note.* IRRs = Incidence Rate Ratios. ^*^*p<* .05, ^**^*p<* .01, ^***^*p<* .001
