# Supplementary material for: Exposure to the 1959–1961 Chinese famine and risk of non-communicable diseases in later life: A life course perspective
Source: PLOS Glob Public Health. 2023 Aug 16;3(8):e0002161. doi: 10.1371/journal.pgph.0002161 (PMC10431657; doi:10.1371/journal.pgph.0002161)
Supplement: Attachment — Submitted filename: Response to Reviewers - 20230531.docx [file pgph.0002161.s008.docx]

Dear Prof. Dr. Sharma,

**Re: “Exposure to the 1959-1961 Chinese famine and risk of non-communicable diseases in later life: A life course perspective” (ref: PGPH-D-23-00113)**

We would like to thank you and the reviewers for your helpful suggestions regarding our manuscript.

Please find our point-by-point responses below. We have responded to each comment provided by you and the reviewers in text boxes, and also included a label for each comment. For your convenience, our responses include all changed sections of the text (with changes highlighted in blue).

We feel that the comments provided by yourself and the reviewers have improved the manuscript substantially, and we hope that it will now meet your criteria for publication.

Thank you again for your time and consideration,

Sincerely,

Mengling Cheng

(on behalf of co-authors)

# **EDITOR’S COMMENTS**

## **E_1_ – Reflect Limitations of Previous Studies, Clarify Study Design, Add Sampling Method, and Add Data Collection**

Thank you for submitting your manuscript to PLOS Global Public Health. After careful consideration, we feel that it has merit but does not fully meet PLOS Global Public Health’s publication criteria as it currently stands. Therefore, we invite you to submit a revised version of the manuscript that addresses the points raised during the review process.

Thank you for your interest in our manuscript and the time you have dedicated to its review. Below is a summary of revisions made to address the concerns.

**1. Limitations of previous studies.** We clarified the limitations of previous studies that we overcame (i.e., the lack of a life course perspective and information bias). We also acknowledge an extra limitation that we were unable to overcome (i.e., survivor bias) and provided possible solutions (Banack et al., 2019). For the complete response to the Reviewer and the revised sections of the text, please see **R1_4_**.

**2. Study design.** We presented our study design more clearly in the Methods and the Abstract. Our study design is now presented as a secondary analysis of a large-scale, nationally representative, longitudinal study, which provides both prospective data and retrospective data: the China Health and Retirement Longitudinal Study (**R2_1_**).

**3. Sampling method.** We referred to the Cohort Profile (Zhao et al., 2012) and provided details of the sample method used in CHARLS (**R2_2_**).

**4. Data collection.** We incorporated information about the data collection approach prior to discussing the study findings and implications (**R1_6_**).

In the course of our revision work, we also decided to provide additional evidence to strengthen our claim that the observed famine effects were independent of age/cohort differences and that the most affected participants were most likely to die in the short or medium term.

In **Overview of the study**, we added the following (**pp. 8-9**):

Moreover, we used more robust methods to adjust for age/cohort and used a more direct measure of famine exposure/severity.

In the **Discussion**, we added the following (**pp. 27-28**):

First, consistent with the few existing studies [37] [22], our analysis using the concatenation approach revealed that individuals exposed to famine at different life stages were affected differently—and the famine effects were independent of age/cohort differences (see **Table S5**).

Second, […] We recognize that we had only retrospective and imperfect measures of famine exposure severity, and famine exposure severity might not be fully captured by the available data (e.g., we had no measure of an individual’s nutritional/anthropometric status at the time of famine). Hence the true effects may have been underestimated. It is also possible that “healthy survivor” bias again applies and that those most affected were most likely to die in the short or medium term (see **Table S6**).

# **REVIEWER #1 (R1)’s COMMENTS**

## **R1_0_ – Opening Comment**

Generally, the paper is supremely written with clearly explained methods, and statistical approaches seem to fit the objectives and the identified gaps. In addition, the study hypothesis and key messages are presented clearly and concisely. The relevance of the study to research, practice, and policy is well emphasized in the key messages section.

Thank you for your encouraging comment and for your time and effort spent providing suggestions. Below is a point-by-point response to your comments.

## **R1_1_ – Improve the Sentence Structure in the Abstract**

The introduction in the abstract is well written, but the sentence structure could be improved for clarity. For example, the sentence “To add to the literature and overcome some of these limitations, we adopted a life course perspective and used more robust methods to investigate the association between exposure to the 1959-1961 Chinese famine and later-life NCDs and if this association depends on: life stage (age) at exposure, severity of exposure, and sex” could be split into two sentences for better readability.

Following your comment, we have restructured the sentence and split it into two sentences. We believe that the changes in the sentence structure provide better readability.

The **Abstract** now reads (**p. 2**):

[…] To add to the literature and overcome some of these limitations, we adopted a life course perspective and used more robust methods. We investigated the association between exposure to the 1959-1961 Chinese famine and later-life NCDs and if this association depends on: life stage at exposure, famine severity, and sex.

## **R1_2_ – Improve the Paragraph Structure**

The 4^th^ sentence of the first paragraph of the introduction seems to introduce a different concept from the paragraph’s main idea. Making a separate paragraph would help.

In response to your comment, in our initial submission, we started the fourth sentence of the introduction in a separate paragraph.

## **R1_3_ – Clarify the Sentence**

The last sentence of the second paragraph starts with “these are”. Since more than one aspect is described in the immediate context, it would be better if “these are...” is precisely defined.

Following your comment, we have clarified this sentence by linking it to the previous sentence.

The relevant sentences in the **Introduction** now read (**p. 6**):

Socioeconomic explanations include life-long consequences of early-life poverty: a greater risk of undernutrition in childhood leads to impaired development, suboptimal educational achievement and earning, and loss of full human potential [10, 11]. These consequences of early-life poverty are associated with poverty in adulthood, which independently increases the risk of NCDs in later life [12].

## **R1_4_ – Clarify and Reflect the Limitations of Previous Studies**

Under the “Overview of the Study”, there is a mention of “...to add to the literature and overcome some of the limitations”. Please, precisely clarify these limitations and how they were overcome. Consequently, show the limitations you failed to overcome and suggest how they should be overcome. Moreover, this failure to overcome these other limitations should be reflected in the conclusions and recommendations.

In response to your comment, in the overview of the study, we have clarified the limitations that we overcame (i.e., the lack of a life course perspective and information bias). In addition, in the discussion, we have acknowledged an extra limitation that we were unable to overcome (i.e., survivor bias) and cited one paper which provides possible remedies to this issue (Banack et al., 2019).

The **Overview of the study** now reads (**pp. 8-9**):

The current study of a large-scale, nationally representative, longitudinal study (≈ 26,000 participants) aimed to add to the literature and overcome some of the limitations found in previous research, that is, the lack of a life course perspective and information bias. Specifically, we adopted a life course perspective by focusing on a wide period of life ranging from in-utero to adulthood (24-40 years) and considering the age at famine exposure. Moreover, we used more robust methods to adjust for age/cohort and used a more direct measure of famine exposure/severity.

In the **Discussion**, we added the following (**pp. 28-29**):

We acknowledge four main limitations. […] Last, survivor bias might be part of the story. Future studies need to evaluate the effects of survivor bias by investigating the possible selection mechanisms and adjust the effect estimates of famine exposure [48].

## **R1_5_ – Rephrase the Objectives**

Under the “Overview of the Study”, “technically, avoid writing objectives in the form of questions”. Please, change them into statements.

Following your comment, we have rephrased our aim and objectives into statements.

The **Overview of the study** now reads (**pp. 8-9**):

[…] Our overall aim was to understand the link between exposure to the Chinese famine of 1959-1961 and NCDs 50 years later in 2011-2018. Our objectives were to determine if the link between famine exposure and NCDs in later life varied among individuals exposed at different life stages, individuals exposed to different levels of famine severity, and women and men.

## **R1_6_ – Add the Data Collection Approach in the Conclusion**

The first sentence of the conclusion should be revised. It should reflect the data collection approach used.

In response to your comment, in the conclusion we have incorporated information about the data collection approach prior discussing the study findings and implications.

The **Conclusion** now reads (**pp. 29-30**):

Our research analyzed a large-scale, nationally representative, longitudinal study, which provides both prospective data and retrospective data: the China Health and Retirement Longitudinal Study (CHARLS, approximately 26,000 participants, 2011-2018, aged 45 and above). Our findings show that famine exposure has long-term detrimental consequences for later-life NCDs and affects individuals exposed at different life stage ranging from in-utero until young adulthood. These findings suggest that […]

## **R1_7_ – Correct Grammatical Issues in Several Sentences**

The following sentences had some grammatical errors, and below are suggested corrections:
• Existing literature suggests that undernutrition in childhood has immediate detrimental and long-term adverse health consequences, including increasing the risk of NCDs in later life.
• Life course epidemiology suggests that the timing of exposure is critical and that the effect of exposure during a specific period may differ from ……
• Thus, individuals exposed to famine at different life stages may be affected differently.
• A major source of information bias is the misclassification of famine exposure ……
• We measured famine exposure and severity of famine exposure using the individual self-reported famine experience, life stages using the age at famine exposure, and later-life health using the number of NCDs. We built a series of Poisson growth curve models to investigate the association between famine exposure and later-life NCDs.
• Regarding the severity of famine exposure (predictor), we used the responses to the questions following up the famine exposure question to build a variable differentiating participants who were unexposed to famine……
• We conducted two sets of sensitivity analyses to test the robustness of the associations between famine exposure, life stage, and later-life health.
• The results observed above were in the sensitivity analysis that used two alternative reference dates (**Table S1**) and an alternative scheme of life stages based on Erikson’s developmental stages (**Table S2**).
• However, the difference in the risk of NCDs in later life between moderately exposed participants and those severely exposed to famine failed.
• We addressed the problem of information bias by using a more direct measure of famine exposure and severity of famine exposure. We also extended existing studies by testing whether this association depends on the life stage at exposure, the severity of famine exposure, and sex. We observed three main findings: ……
• Second, we could not control for birth weight or gestational age, which might be important confounders because information on these is unavailable.
• However, several studies showed that the association between famine exposure and NCDs remains after controlling for birth weight.
• These findings suggest that in an individual’s life course, in-utero and the “first 1,000 days” is a particularly sensitive time window of development and growth but that this time window remains open until young adulthood.

We have carefully read the text and corrected the grammar issues.

## **R1_8_ – Correct the Placement of Punctuation Mark**

Lastly, the correct placement of punctuation mark.

Following your comment, we have carefully read the text and corrected the placement of punctuation mark.

# **REVIEWER #2 (R2)’s COMMENTS**

## **R2_0_ – Opening Comment**

This is an excellent study. Very concise yet informative. The authors left no room for redundancy. There is the logical flow of ideas. The introduction is well written. The gap and justification for this study was also well stated. The authors identified limitations and methodological errors/biases in previous studies which is very critical for the understanding and readers’ appreciation of this current study. I love the fact that, the authors identified all the potential confounding factors right from the beginning of the study and controlled for them.

Thank you for your time and effort spent providing suggestions. Below is a point-by-point response to your comments.

## **R2_1_ – Add the Study Design in the Methods**

The study design used was not clear or mentioned in the methods section until at the tail end of the manuscript (during their discussion of study limitations) which leaves readers wondering for long.

In response to your comment, we have added the study design in the methods. In addition, we have added the study design in the abstract. We believe these changes make our study design clear for readers.

In the first paragraph of the **Methods**, we added the following (**p. 9**):

We conducted a secondary analysis of a large-scale, nationally representative, longitudinal study, which provides both prospective data and retrospective data: the China Health and Retirement Longitudinal Study (CHARLS), which followed Chinese urban and rural residents aged 45 years old and above from 2011 to 2018. We measured […]

In the **Methods**, we added the following (**pp. 10-11**):

**Data and participants**

We used the data from the CHARLS, a large-scale, nationally representative, longitudinal study that provides both prospective data and retrospective data. CHARLS collects prospective health data from Chinese urban and rural residents aged 45 years old and above biennially since 2011. […] In our analyses, we merged the retrospective Life History data collected in 2014 with all four regular data waves collected in 2011, 2013, 2015, and 2018 (25,863 participants).

In the **Abstract**, we added the following (**p. 2**):

[…] We conducted a secondary data analysis of a large-scale, nationally representative, longitudinal study—the China Health and Retirement Longitudinal Study (2011-2018, 11,094 participants).

## **R2_2_ – Add the Sampling Method**

The sampling method and technique used in the selection of the sample population. I see a flow diagram describing reasons for excluding certain participants and the final sample size used. But that still does not clearly answer the question on the sampling method used. This should have been briefly discussed in the methods section.

In response to this insightful suggestion, we have revisited the Cohort Profile (Zhao et al., 2012) and provided details of the sample method used in CHARLS.

In the **Methods**, we added the following (**p. 10**):

**Data and participants**

[…] CHARLS applies a stratified, multistage probability sampling design [29]. First, CHARLS uses a probability proportion to size (PPS) sampling approach for all county-level unit except for Tibet after stratification by region, county characteristic (urban or rural), and per-capita gross domestic product. Primary sampling units (PSUs) are administrative villages in rural areas and neighborhoods in urban areas. Second, CHARLS uses CHARLS-GIS software to randomly sample households within each PSU. Last, within each sampled household, if occupants were aged above 40, one of them is randomly selected. If the selected person was aged 45 or above, they become the main respondent and their spouse is also interviewed; if the selected person was aged between 40 and 44, they are reserved as a refreshment sample. If an age-eligible person was too frail to answer questions, a proxy respondent is identified to help them to answer questions. The sample is representative of participants aged 45 and above living in households.
