# Supplementary material for: Exposure to the 1959–1961 Chinese famine and risk of non-communicable diseases in later life: A life course perspective
Source: PLOS Glob Public Health. 2023 Aug 16;3(8):e0002161. doi: 10.1371/journal.pgph.0002161 (PMC10431657; doi:10.1371/journal.pgph.0002161)
Supplement: Attachment — Submitted filename: Review PLOS.docx [file pgph.0002161.s009.docx]

**Review**

**PGPH-D-23-00113R1: Exposure to the 1959-1961 Chinese famine and risk of no-communicable diseases in later life: A life course perspective**

I have had the opportunity to thoroughly review the article titled " **Exposure to the 1959-1961Chinese famine and risk of no-communicable diseases in later life: A life course perspective**. I am pleased to provide my feedback and highly recommend its publication in the esteemed Journal of PLOS Global Public Health.

The article is exceptionally well written, demonstrating a profound understanding of the subject matter and presenting a comprehensive analysis. The methodology employed is rigorous, the data analysis and interpretation are thorough, with clear and concise descriptions of statistical methods used. The findings are presented in a logical manner, effectively supporting the article's main arguments. I believe that publishing it in this journal would greatly contribute to the advancement of knowledge in the field.
